# Assessing behavioral and psychological symptoms of dementia: a comprehensive review of current options and future perspectives

**DOI:** 10.3389/frdem.2023.1226060

**Published:** 2023-07-25

**Authors:** Federico Emanuele Pozzi, Luisa Calì, Carlo Ferrarese, Ildebrando Appollonio, Lucio Tremolizzo

**Affiliations:** ^1^Department of Neurology, Fondazione IRCCS San Gerardo dei Tintori, Monza, Italy; ^2^Milan Center for Neuroscience (NeuroMI), University of Milano-Bicocca, Milan, Italy; ^3^School of Medicine and Surgery, University of Milano-Bicocca, Milan, Italy

**Keywords:** BPSD, dementia, assessment, scales, behavior

## Abstract

The behavioral and psychological symptoms of dementia (BPSD) are a heterogeneous set of challenging disturbances of behavior, mood, perception, and thought that occur in almost all patients with dementia. A huge number of instruments have been developed to assess BPSD in different populations and settings. Although some of these tools are more widely used than others, no single instrument can be considered completely satisfactory, and each of these tools has its advantages and disadvantages. In this narrative review, we have provided a comprehensive overview of the characteristics of a large number of such instruments, addressing their applicability, strengths, and limitations. These depend on the setting, the expertise required, and the people involved, and all these factors need to be taken into account when choosing the most suitable scale or tool. We have also briefly discussed the use of objective biomarkers of BPSD. Finally, we have attempted to provide indications for future research in the field and suggest the ideal characteristics of a possible new tool, which should be short, easy to understand and use, and treatment oriented, providing clinicians with data such as frequency, severity, and triggers of behaviors and enabling them to find appropriate strategies to effectively tackle BPSD.

## 1. Introduction

The behavioral and psychological symptoms of dementia (BPSD) are a heterogeneous set of signs and symptoms of disturbed perception, thought content, mood, or behavior (Finkel et al., 1998). The prevalence of individual BPSD varies between 10% and 20%, but up to 90% of people living with dementia will have at least one, depending on the setting (Cerejeira et al., 2012; Kwon and Lee, 2021; Altomari et al., 2022). A few BPSD also have been included in the diagnostic criteria of specific dementias (Rascovsky et al., 2011; McKeith et al., 2017), and they are already frequently found in the prodromal phases (Pocnet et al., 2015; Van der Mussele et al., 2015; Wiels et al., 2021), sometimes even being the main presenting symptom in the preclinical condition called mild behavioral impairment (MBI) (Creese and Ismail, 2022). Various attempts have been made to cluster them into broader categories, such as psychosis, affective syndrome, apathy, mania, and psychomotor syndrome, but a consensus on the topic is lacking (D'antonio et al., 2022). Perhaps due to their elusive nature in terms of both definition and rigorous assessment, these non-cognitive symptoms, in spite of their enormous prevalence, have often been overshadowed by the various sets of diagnostic criteria for dementia syndrome, in general, and Alzheimer's disease, in particular, both of which mainly focus on the cognitive aspects of the two conditions (Gottesman and Stern, 2019).

Moreover, BPSD have a huge impact on persons living with dementia, caregivers, and healthcare systems, in terms of both quality of life and economic burden (Cerejeira et al., 2012). Current guidelines recommend the use of non-pharmacological interventions as a first line of treatment, although there is no consensus on what these interventions should entail (Azermai et al., 2012; Ma et al., 2022). In clinical practice, the use of antipsychotics remains common, although the effectiveness of most of them is still debated and their use may result in serious adverse events (Kales et al., 2014). This may also be partially due to the fact that BPSD are traditionally assessed with scales that are unsuitable for non-pharmacological interventions, and triggers of BPSD are rarely systematically explored (Kales et al., 2014). However, in new approaches, there have been attempts to include these aspects (Kales et al., 2014; Tible et al., 2017) and even to anticipate the future occurrence of BPSD with the aid of contextual screening (Evans et al., 2021). In a comprehensive review published a decade ago, more than 80 scales for BPSD assessment in different populations and settings were discovered, although not all of them were judged to be specific to patients with dementia (Van derlinde et al., 2014). Since then, the number of tools that clinicians can use has further increased, and new approaches have been introduced, resulting in a plethora of instruments from which clinicians can choose to frame the complex issue of behavioral problems in persons with dementia. Focusing on the measurements of BPSD is important since a better understanding of the problem is fundamental for guiding rational strategies to address it. Therefore, we suppose that a study that provides information to orient the choice of tool in specific contexts is needed.

The aim of the current study is to provide an updated overview of the instruments currently available for clinicians to evaluate BPSD in patients with dementia, together with the advantages and disadvantages of the different approaches.

Theoretical frameworks have been developed to explain BPSD from different points of view: the biomedical approach regards behaviors as neurocognitive symptoms, and the biopsychosocial view seeks to understand the behaviors by analyzing all possible person-centered and environmental contributors (Nagata et al., 2022). While our perspective in organizing this study may rely more on the former approach, we are not insensitive to the latter, and indeed, we recognize that combining the two views can help understand and address BPSD more effectively.

## 2. Methods

The scales and approaches included in our study were located through a non-systematic search of the major databases (including MEDLINE via PubMed and Scopus), a citation search on the articles included, and personal knowledge. While this does not exclude the possibility that some instruments have been left out, we presume that such tools would not have a great impact on the field, as they would have been mentioned somewhere in our extensive list of references.

We specifically excluded those scales that have been adapted from other contexts and conditions, such as psychiatric diseases (such as the state-trait anxiety inventory and the Hamilton depression scale) or that are intended as global screening tools for dementia (such as the Blessed dementia scale) and focused only on instruments that have been developed to capture behavioral disturbances in patients with cognitive impairment. We also excluded screening checklists, which can, however, be useful to identify unmet needs for specialized care, such as the BEHAV5+ checklist, which assesses the presence of six behavioral issues and is endorsed by the Alzheimer's Association within the Cognitive Impairment Care Planning Toolkit (Borson et al., 2014).

The present study is organized into four major sections. The first describes the characteristics of the instruments available for the assessment of BPSD, dividing them into broad and narrow tools, evaluating the population, the setting, the timing, and the rater, and considering their uses (paragraphs 3.1–3.4). Information on different translations used in research on the use of BPSD tools was retrieved by checking in Scopus the list of works that have cited the original paper for each instrument (paragraph 3.5). While this could leave out possible versions included in articles not indexed in Scopus or not used in any study at all, we believe that our approach would increase the reliability of the translations themselves. BPSD have been grouped into categories reflecting the 12 items of the Neuropsychiatric Inventory (NPI-12), probably the most common instrument used in clinical practice. However, we decided to separate physically and verbally aggressive behaviors, as these are often treated differently and may have different impacts on treatment choices. We also added three more categories: “harmful to self” and “uncooperativeness”, which would be proportionally more relevant in particular settings such as nursing homes and “other” to include all behaviors that do not fit into the aforementioned BPSD (such as toileting problems or hiding things). A list of possible synonyms used to categorize BPSD is presented in Table 1.

**Table 1:** Examples of synonyms used to categorize single items of the scales into recognizable BPSD.

**Category**	**Possible synonyms**
Aberrant motor behavior	Trying to leave home, wandering, rummaging, pacing, increased motor activity, fiddling, exit seeking, purposeless activity
Agitation	Restlessness, fidgetiness, screaming, crying out loud, inability to sit still, attention seeking, complaining, and repetitive behavior or questions
Aggression, physical	Physically threatening and destroying property
Aggression, verbal	Raising the voice, quarreling with others, cursing, shouting, and demanding
Anxiety	Worrying, fearfulness, phobias, panic attacks, and fear of being left alone
Apathy	Lack of energy, aspontaneity, and indifference
Delusion	False accusations, disease denial or distortion, non-sense, and paranoia
Depression	Tearfulness, sadness, negativism, and anhedonia
Disinhibition	Socially inappropriate behaviors, sexually inappropriate behaviors, and impulsivity
Eating	Pica, dietary changes, appetite loss, and hyperphagia
Euphoria	Excitation and mania
Hallucinations	Olfactory hallucinations, visual hallucinations, auditory hallucinations, and feeling of a presence
Harmful to self	Dangerous behavior, suicidal, and intentional falling
Irritability	Arguing, complaining, and impatience
Sleeping problems	Day/night reversal, day/night disturbances, being noisy at night, and wandering at night
Uncooperativeness	Refusing help and uncooperativeness to testing

In the second section, we have discussed the methodological aspects of the scales that relate to their validity and reliability, which we presume are fundamental to understanding current pitfalls in the development of new instruments (paragraph 3.6), while the third section briefly focuses on new approaches, such as treatment-oriented approaches, and on actigraphy and other objective biomarkers of BPSD (paragraphs 3.7–3.8). Finally, we attempt to translate these data from the literature into indications for future research (paragraph 3.9).

## 3. Results

### 3.1. Instruments focusing on a broad range of BPSD

For the purpose of the current classification, we have arbitrarily defined broad instruments as scales encompassing more than four BPSD. Broad instruments are summarized in Table 2.

**Table 2:** Broad instruments—number of items per BPSD.

**Scale**	**Items**	**Abe**	**Agi**	**AP**	**AV**	**Anx**	**Apa**	**Del**	**Dep**	**Dis**	**Eat**	**Eup**	**Hal**	**Irr**	**Sle**	**Unc**	**HTS**	**Other BPSD**
ABID; Logsdon et al. (1999)	16	1	3	2	1	1	-	1	-	2	-	-	1	1	1	1	1	-
ABMI; Cohen-Mansfield et al. (1989a)	14	3	5	2	2	-	-	1	-	-	1	-	1	-	-	-	-	-
ABS; Abe et al. (2015)	10	1	1^*^	1	1	-	1	1^$^	1	-	1^§^	1^*^	1^$^	1	1	-	-	1^§^, toileting problems
ACID; Gerolimatos et al. (2015)	13	-	1	-	-	3	-	-	-	-	-	-	-	1	1	-	-	7, aspecifically anxiety related
ADAS-Ncog; Rosen et al. (1984)	10	2	-	-	-	-	-	1	2	-	1	-	1	-	-	1	-	2, distractability and tremors
BEAM-D; Sinha et al. (1992)	18	1	1	2	1	1	1	1	1	1	1	1	1	1	1	2	-	4, disruption of others' activities, hoarding, appropriateness, and stability of affect
BEHAVE-AD; Reisberg et al. (1987)	25	3	1	1	1	4	-	7	2	-	-	-	5	-	1	-	-	-
BMDS; Greene et al. (1982)	34	3	3	1	1	1	8	1	2	-	-	-	-	1	2	-	1	10, hoarding and cognitive aspects
BPC; O'Malley and Qualls (2016)	34	-	-	1^*^	1^*^	1	2	-	3	1	2	-	-	1	1	-	1	21, personality changes, suspiciousness, social relations, energy, homicidal thoughts, other cognitive aspects, and 2 “other” items
BPSD-DS; Dekker et al. (2018)	12	-	1	1^*^	1^*^	1	1	1	1	1	1	-	1	1	1	-	-	1, obstinacy
BPSD-T; Phannarus et al. (2020)	14	2	-	1^*^	1^*^	1	1	1	1	-	-	1	1	1	1	-	-	3, repeating, misidentification, and hoarding
BSO-DOS; BSO-DOS (2019)	8+	1	1	1	1	-	-	-	-	1	-	-	-	-	-	-	-	Possibility of tracking other behaviors
BSSD; Devanand et al. (1992a)	30+	1	1	1	1	1	7	2	1	3	-	1	-	1	-	-	1	7, denial, sundowning (x2), self-centeredness, stubbornness, dependency, and stereotypy
CABOS; Burgio et al. (1994)	1	-	1^*^	-	1^*^	-	-	1^*^	1^*^	1^*^	-	-	-	-	-	-	-	-
CAMDEX; Roth et al. (1986)	?	1	-	-	-	-	-	1	21	1	-	-	-	-	-	-	-	-
CBS; Moniz-Cook et al. (2001)	25	1	5	1	1	-	2	-	-	4	-	-	-	-	1	1	1	8, clinging, interfering with other people, hoarding, suspiciousness, manipulative, lack of self-care, fecal smearing, and dangerous behavior
CBS-8; Mansbach et al. (2021)	8	1	1^*^	1	1	1^*^	-	1	-	1	-	-	1	1	-	-	-	-
CDBQ; Victoroff et al. (1997)	81	3	5	2	1	3	7	12	7	3	4	1	10	2	4	1	-	14, hoarding, shadowing, and other cognitive aspects
CERAD/BRSD; Tariot et al. (1995)	48	3	3	1	1	2	3	8	7	1	2	-	5	1	2	1	2	6, tiredness, change in sexual drive, sudden change in emotion, somatic complaints, daily pattern of confusion, and other unspecified
CMAI; Cohen-Mansfield et al. (1989b)	29	3	7	8^*^	1	-	-	-	1	3	-	-	-	-	-	-	3^*^	3, hoarding, hiding, and handling things
COBRA; Drachman et al. (1992)	30	4	2	3	2	-	1	3	-	1	1	-	2	-	1	1	1	7, incontinence, weight change, personality change, pain tolerance, bradykinesia, hoarding, reclusiveness, and following
CPCE; Resnick et al. (2018)	8	1	3	1	-	-	1	-	-	1	-	-	-	-	-	1	-	-
CSDD; Alexopoulos et al. (1988)	19	-	1	-	-	1	1	1	5	-	1	-	-	1	3	-	1	4, retardation, multiple physical complaints, weight loss, and diurnal variation
CUSPAD; Devanand et al. (1992b)	24	1	1	1	1	-	-	10	1	-	1	-	6	-	1	-	-	1, sundowning
DBDS; Baumgarten et al. (1990)	28	3	4	2	1	-	1	1	-	4	2	-	-	-	3	1	-	6, getting lost outside, hoarding, hiding, incontinence (x2), and throwing food
DBRI; Molloy et al. (1991)	25	2	3	1	1	1	1	5	-	1	-	-	2	2	1	1	-	4, repeating stories, being frustrated, keeping changing mind, and hiding things
DBS; Beck et al. (1997)	45	2	5	14	1	-	-	-	-	10	2	-	-	-	-	1	2	8, isolating from others, losing track of own objects, taking objects from others (x2), spitting medication, talking constantly, throwing food, and not maintaining personal hygiene
DMAS; Sunderland et al. (1988)	17	-	1	-	-	1	2	-	5	-	1	-	-	1	1	-	1	4, psychosomatic complaints, energy, awareness of emotional status, and speech
DSSS; Loreck et al. (1994)	43	1	3	1	1	3	2	8	5	2	1	4	4	1	2	1	-	4, somatic complaints, following, unusual behavior, and unsafe behaviors
HBS; Draper et al. (2002)	23	-	-	2	1	-	-	-	1	-	1	-	-	-	-	6	7	5, refusing to take part in social activities, alienating staff, burning, other, and overdosing medication
MBI-C; Ismail et al. (2017)	34	-	2^*^	1^*^	1^*^	2	6	3	4	8	2	-	2	3^*^	-	-	-	3, hoarding, stubbornness, and a lack of empathy
MOUSEPAD; Allen et al. (1997)	59	3	2	6^*^	5^*^	-	-	22	-	2	4	-	15	-	4	-	-	3, shadowing, hiding, and mislaying
NHBPS; Ray et al. (1992)	29	2	5	2	1	-	-	2	-	3	-	-	-	1	3	4	3	2, asking or complaining about health, complaining, or whining
NPI; Cummings et al. (1994)	10	1	1^*^	1^*^	1^*^	1	1	1	1	1	-	1	1	1	-	-	-	Eating and sleeping in NPI-12
PBE; Hope and Fairburn (1992)	121+	28	?	1	1	?	?	?	?	5	30	?	?	?	?	?	?	Y, many others
RAGE; Patel and Hope (1992)	21	-	2	7	2	-	-	-	-	-	-	-	-	2	-	2	1	4, critical, antisocial acts, angry with self, and global impression
RMBPC; Teri et al. (1992)	24	-	-	2	2	1	-	-	8	1	-	-	-	1	1	-	-	8, memory (x7) and hiding things
TBS; Asada et al. (1999)	14	2	2	1^*^	2^*^	-	-	2	-	-	1	-	-	-	1	-	1	3, interfering with a happy home circle, making the dwelling dirty, and hiding
WCA; Kales et al. (2017)	?	?	?	?	?	?	?	?	?	?	?	?	?	?	?	?	?	Toileting

BPSD that are grouped as single items have the same symbol (such as ^*^; § or $). When no information on the exact number of items could be retrieved; a question mark (?) was placed under the corresponding column.

Abe, aberrant motor behavior; Agi, agitation; AP, physical aggression; AV, verbal aggression; Anx, anxiety; Apa, apathy; Del, delusions; Dis, disinhibition; Eat, eating; Eup, euphoria; Hal, hallucination; Irr, irritability; Sle, sleeping problems; Unc, uncooperativeness; HTS, harmful toward self.

A huge variety of scales exists, with different numbers of items per BPSD. Among these scales, the most complete is probably the NPI (Cummings et al., 1994), which also has the advantage of reducing redundancy by assigning only one item to each BPSD of interest. In the NPI-12 version, the scale also includes eating and sleeping problems. However, the NPI groups together physical and verbal aggression and agitation, which probably makes it less sensitive to problems that may be worth discriminating. Other instruments inspired by the same principles of comprehensiveness and brevity include the Columbia Behavior Scale for Dementia (CBS-8), the Abe's BPSD score (ABS), and the BPSD in Down syndrome scale (BPSD-DS). The ABS groups together delusion and hallucinations, eating and toileting problems, and agitation and euphoria but discriminates between verbal and physical aggression. However, the various items are scored differently, with proportionally more weight given to aberrant motor behavior and eating and toileting problems (Abe et al., 2015). The CBS-8 groups together agitation and anxiety, which may be harder for caregivers to distinguish, and does not register behaviors that are less disruptive, such as apathy and depression (Mansbach et al., 2021). The BPSD-DS (Dekker et al., 2018), such as the NPI, does not differentiate between physical and verbal aggression.

Almost all other broad instruments consider at least one BPSD with more than one item. When the scale is intended to be used with a cumulative score, this is equivalent to giving weights to single dimensions, and therefore, in most cases, the scale is oriented toward a certain cluster (or clusters) of BPSD. Some of these scales are more focused on “productive” behaviors. For instance, the Disruptive Behavior Scale (DBS) assigns proportionally more weight to physical aggression and disinhibition, with 14 and 10 items out of a total of 45 items pertaining to these categories (Beck et al., 1997). The Rating Scale for Aggressive Behavior in the Elderly (RAGE) uses nine items out of 21 to investigate aggression (Patel and Hope, 1992). The California Dementia Behavior Questionnaire Caregiver and Clinical Assessment of Behavioral Disturbance (CDBQ) assigns 12 items to delusion and 10 to hallucinations of a total of 81 items (Victoroff et al., 1997). The Columbia University Scale for Psychopathology in Alzheimer's disease (CUSPAD) follows in the same direction by assigning two-thirds of the items to delusions and hallucinations (Devanand et al., 1992a). The Manchester and Oxford Universities Scale for the Psychopathological Assessment of Dementia (MOUSEPAD) goes even further, with roughly half of its 59 items pertaining to the psychotic cluster (Allen et al., 1997). The Cohen-Mansfield Agitation Inventory (CMAI), which is intended as a measure of agitation, gives proportionally more weight to agitation and physical aggression but still includes a broad range of BPSD, such as aberrant motor behavior, disinhibition, and even depression (Cohen-Mansfield et al., 1989b).

In contrast, other scales focus more on “negative” behaviors, such as apathy or depression. For example, the Behavioral and Mood Disturbance Scale (BMDS) gives a relatively high weight to apathy, with eight items out of 34. However, this scale also includes 10 items related to cognitive aspects, which makes it less specific to the evaluation of BPSD (Greene et al., 1982). The Dementia Mood Assessment Scale (DMAS) and the Revised Memory and Behavior Problems Checklist (RMBPC) assign roughly one-third of their respective items to depression (Sunderland et al., 1988; Teri et al., 1992). Finally, the Cornell Scale for Depression in Dementia (CSDD), despite its name, includes a huge variety of BPSD, with only a quarter of the items properly pertaining to depression (Alexopoulos et al., 1988). It is clear that the choice of instrument implies different kinds of overall judgments on the degree of the patient's behavioral problems, and the specific characteristics of the tool need to be kept in mind when applying it in clinical practice.

Another common weakness of this second group of broad instruments is that they generally include a very high number of items (the Present Behavioral Examination [PBE (Hope and Fairburn, 1992)] contains more than 120 questions). This does not necessarily translate into a better characterization of the patient's problems, since the extreme parcellation of psychopathology this requires may, in fact, be limited by the ability of raters to differentiate between similar behavioral manifestations. Moreover, for practical rather than research purposes, such a level of detail might be completely useless and not worth the time spent acquiring the required information.

Other broad instruments evaluate BPSD in the context of a more comprehensive assessment that also includes cognitive aspects. This may be done by classifying BPSD as a specific separate section of a larger instrument, as is the case with the Cambridge Mental Disorders of the Elderly Examination (CAMDEX) (Roth et al., 1986) or the Alzheimer's Diseases Assessment Scale (ADAS-Noncog) (Rosen et al., 1984), or by mixing cognitive and behavioral items in the instrument, as in the Consortium to Establish a Registry for Alzheimer's Disease/Behavior Rating Scale for Dementia (CERAD/BRSD) (Tariot et al., 1995), the CDBQ (Victoroff et al., 1997), or the RMBPC (Teri et al., 1992).

Finally, for broad instruments, a single score may not be relevant at all for clinical or even research purposes, as it will not be able to discriminate between dramatically different types of patients. For instance, a patient with isolated severe apathy and a patient with important isolated aggressive behaviors may have the same, rather low score of 12 on the NPI. However, these are two completely different patients, who would require substantially contrasting approaches. A few instruments, such as the Behavioral Syndromes Scale for Dementia (BSSD), attempted to circumvent this issue by allowing the use of subscores for each cluster (Devanand et al., 1992b). The only reason for using broad instruments with a single summary score would be to give the clinician an idea of the amount of behavioral disturbances of a patient at a certain time point to evaluate the need for specific interventions and their efficacy, by administering the same scale at a second time point. However, qualitative data still need to be retained, and the scores cannot be used in an acritical manner.

### 3.2. Instruments focusing on specific BPSD

Narrow instruments are summarized in Table 3.

**Table 3:** Narrow instruments—number of items per BPSD.

**Scale**	**Items**	**Abe**	**Agi**	**AP**	**AV**	**Anx**	**Apa**	**Del**	**Dep**	**Dis**	**Eat**	**Eup**	**Hal**	**Irr**	**Sle**	**Unc**	**HTS**	**Other BPSD**
ABC-DS; Kikuchi et al. (2018)	3	-	1	-	-	-	-	-	-	-	-	-	-	1	-	1	-	-
ABeS; Perlman and Hirdes (2008)	4	-	-	1	1	-	-	-	-	1	-	-	-	-	-	1	-	-
Apathy Inventory; Robert et al. (2002)	3	-	-	-	-	-	3	-	-	-	-	-	-	-	-	-	-	-
AWS; Algase et al. (2001)	29	29	-	-	-	-	-	-	-	-	-	-	-	-	-	-	-	
BADGP; Ferm (1974)	13	-	1	-	-	-	1	-	-	-	-	-	-	-	1	-	-	10, cognitive aspects
BARS; Finkel et al. (1993)	10	1	6	3	-	-	-	-	-	-	-	-	-	-	-	-	-	-
DAIR; Strauss and Sperry (2002)	16	-	-	-	-	-	16	-	-	-	-	-	-	-	-	-	-	-
DBRS; Mungas et al. (1989)	4	1	1	1	1	-	-	-	-	-	-	-	-	-	-	-	-	-
IAS; Burns et al. (1990)	10		-	-	2^*^	-	5	-	-	-	-	-	-	5^*^	-	-	-	-
IQ; Craig et al. (2008)	10 (CIQ)	-	-	3	2	-	-	-	-	-	-	-	-	2	-	-	-	3, withdrawn, critical, frustrated
PAS; Rosen et al. (1994)	4	-	2	1^*^	1^*^	-	-	-	-	-	-	-	-	-	-	1^*^	-	-
PEAR; Jao et al. (2016)	6	-	-	-	-	-	6	-	-	-	-	-	-	-	-	-	-	-
RAID; Shankar et al. (1999)	18	-	3	-	-	6	-	-	-	-	-	-	-	1	1	-	-	7, aspecific autonomic correlates of anxiety
RAS; Ryden (1988)	25	-	-	16	4	-	-	-	-	5	-	-	-	-	-	-	-	-
RTC-DAT; Mahoney et al. (1999)	13	-	-	-	-	-	-	-	-	-	-	-	-	-	-	13	-	-
SDI; Tractenberg et al. (2003)	8	-	-	-	-	-	-	-	-	-	-	-	-	-	8	-	-	-
SOAPD; Hurley et al. (1999)	7	-	7	-	-	-	-	-	-	-	-	-	-	-	-	-	-	-
SSADE; Takada et al. (2017)	11	1^*^	1^*^	-	-	-	-	1^*^	-	-	9^*^	-	-	-	-	1^*^	-	2, dietary agnosia and eating apraxia

BPSD that are grouped as single items have the same symbol (^*^).

Abe, aberrant motor behavior; Agi, agitation; AP, physical aggression; AV, verbal aggression; Anx, anxiety; Apa, apathy; Del, delusions; Dis, disinhibition; Eat, eating; Eup, euphoria; Hal, hallucination; Irr, irritability; Sle, sleeping problems; Unc, uncooperativeness; HTS, harmful toward self.

The Algase Wandering Scale (AWS) was the only tool specifically designed to investigate taberrant motor behavior in patients in long-term care, with 28 items divided into a number of subscales (Algase et al., 2001).

Agitation can be better evaluated by the Brief Agitation Rating Scale [BARS (Finkel et al., 1993)], the Pittsburgh Agitation Scale [PAS (Rosen et al., 1994)], and the Scale for the Observation of Agitation in Persons with Dementia of the Alzheimer's type [SOAPD (Hurley et al., 1999)]. All of these scales have the advantage of being relatively short, with no more than 10 observational items; however, except for the SOAPD, they also include items related to aggression. However, the SOAPD scoring system is, unfortunately, extremely complicated, with weight given to each of its seven items, and this may explain its relatively low level of use. They are all to be used in nursing homes (NH) and rated by nurses; the PAS can also be used to evaluate hospital inpatients, and it is probably the best instrument to rate agitation.

The Ryden Aggression Scale (RAS) is a caregiver-rated instrument focusing specifically on aggression, with 20 items pertaining to aggression and five items more loosely related to disinhibition (Ryden, 1988). The Aggressive Behavior Scale (called as ABeS in this review to distinguish it from Abe's BPSD score, but commonly abbreviated as ABS) has almost the same features, but it has the advantage of being shorter (Perlman and Hirdes, 2008). The Disruptive Behavior Rating Scales (DBRS) are quick instruments focusing on particularly problematic symptoms and include aggression, agitation, and aberrant motor behaviors (Mungas et al., 1989). The ABC Dementia Scale (ABC-DS) has three domains: Domain B (BPSD) includes aspects of agitation, irritability, and uncooperativeness, while the other two domains pertain to activities of daily life and cognition (Kikuchi et al., 2018). The Irritability Questionnaire (IQ), which has two separate forms, one to be administered to the person and one to the caregiver, dedicates half the items in the latter to aggression, while the patient form focuses more properly on irritability (Craig et al., 2008). Other drawbacks of this scale are that it partially relies on the ability of the patient to self-report symptoms, which may be impaired in the later stages of dementia, and the fact that some items appear to be rated in opposite directions, which would make the use of a total score less meaningful. For instance, “I have been feeling relaxed” and “things are going according to plan” are rated the same as “I lose my temper and shout or snap at others”, which may be a mistake.

The Rating Anxiety in Dementia scale (RAID) offers a comprehensive evaluation of anxiety, although a number of items are quite non-specific (such as palpitations or fatigability, which could also be related to general medical conditions) (Shankar et al., 1999).

While no scale is specifically designed to rate depression, a few instruments focus specifically on apathy, such as the Apathy Inventory [probably the shortest one, with only three items (Robert et al., 2002)], the Dementia Apathy Interview and Rating scale [DAIR, only suitable for mild and moderate phases (Strauss and Sperry, 2002)], and the more recent Person–Environment Apathy Rating [PEAR (Jao et al., 2016), see Section 3.6]. Half the items in the Irritability-Apathy Scale (IAS) pertain to apathy and half to irritability, although they clearly do not reflect the same latent variable (Burns et al., 1990).

No tools focus specifically on symptoms such as delusions, hallucinations, or euphoria. Finally, a few scales are available for particular issues, such as the Signs and Symptoms Accompanying Dementia while Eating [SSADE (Takada et al., 2017)], the Sleep Disorders Inventory [SDI (Tractenberg et al., 2003)] and the Resistiveness to Care Scale [RTC-DAT (Mahoney et al., 1999)].

### 3.3. Instruments by populations

The vast majority of tools have been developed for patients with unspecified dementia, although the prevalence of BPSD such as apathy, depression, and anxiety may vary among different forms of neurodegenerative diseases (Collins et al., 2020). Considerably fewer instruments have been validated for particular diseases, the most common being AD. These instruments include the already mentioned ABC-DS, ADAS-Noncog, Apathy Inventory, CUSPAD, SDI, and SOAPD (Rosen et al., 1984; Devanand et al., 1992a; Hurley et al., 1999; Robert et al., 2002; Tractenberg et al., 2003; Kikuchi et al., 2018), and the Troublesome Behavior Scale [TBS (Asada et al., 1999)], the Agitated Behavior in Dementia scale [ABID (Logsdon et al., 1999)], and the Behavioral Pathology in Alzheimer's Disease Rating Scale [BEHAVE-AD (Reisberg et al., 1987)]. Two scales have been tested in mixed populations, with different results, as is the case with the IAS and IQ, which also included patients with Huntington's disease (HD). The IAS shows higher values for aggression for HD patients compared to AD patients (Burns et al., 1990), but neither scales demonstrated significant differences in irritability between the two conditions (Burns et al., 1990; Craig et al., 2008).

A few instruments explore BPSD in specific disorders. The BPSD-DS is intended to be used only in persons with Down syndrome aged over 30 years, and, accordingly, it includes items that are peculiar to the psychopathology of this disease, such as obstinacy (Dekker et al., 2018). The Mild Behavioral Impairment Checklist (MBI-C) is a rather long consensus-derived instrument, specifically envisaged for the recently introduced phase of MBI, in which mild behavioral symptoms analogous to BPSD may predate the onset of cognitive impairment. This scale is designed to comprehensively explore the diagnostic criteria for this condition; in this sense, it may be observed as a diagnostic tool rather than a form of behavioral assessment (Ismail et al., 2017). Subsequent papers introduce different cutoff levels for identifying MBI in subjective cognitive decline (>6.5) and mild cognitive impairment (>8.5) (Mallo et al., 2018, 2019).

Many instruments have been validated in the elderly population, with or without cognitive impairment, especially in the NH context. These instruments mostly focus on agitation and disruptive behaviors that may pertain to a more general population of cognitively impaired and psychiatric elderly patients, including the Agitation Behavior-Mapping Instrument [ABMI (Cohen-Mansfield et al., 1989a)], BARS (Finkel et al., 1993), CMAI (Cohen-Mansfield et al., 1989b), Harmful Behaviors Scale [HBS (Draper et al., 2002)], and RAGE (Patel and Hope, 1992). Another use of assessment scales in the general elderly population is in the diagnostic evaluation and follow-up assessment in outpatient memory clinics of persons who may have cognitive impairment. These instruments, which also include cognitive aspects, are represented by the CAMDEX, DBRI, and BMDS (Greene et al., 1982; Roth et al., 1986; Mungas et al., 1989). Finally, the Behavioral Problem Checklist (BPC) is a scale designed to capture aspects of psychopathology and caregiver distress in patients with dementia or chronic physical illness (O'Malley and Qualls, 2016).

### 3.4. Instruments by setting, timing, and rater

The characteristics of the different tools in terms of population, rater, setting, and timing are summarized in Table 4.

**Table 4:** Instruments by setting.

**Acronym**	**Population**	**Rater**	**Period**	**Setting**
ABC-DS; Kikuchi et al. (2018)	AD patients	Healthcare staff^*^	12 weeks	OC
ABID; Logsdon et al. (1999)	AD patients	Caregiver	Two 1-week intervals	OC
ABMI; Cohen-Mansfield et al. (1989a)	Elderly patients	Nurse	2 weeks	NH
ABeS; Perlman and Hirdes (2008)	Patients with dementia	Nurse	1 week	NH
ABS; Abe et al. (2015)	Patients with dementia	Caregiver	Not stated	Home or NH
ACID; Gerolimatos et al. (2015)	Patients with dementia	Clinician^**^	1 day	OC
ADAS-Noncog; Rosen et al. (1984)	AD patients	Clinician^***^	1 week	OC
Apathy Inventory; Robert et al. (2002)	AD patients	Clinician^**^	Not stated	OC
AWS; Algase et al. (2001)	Patients with dementia	Healthcare staff	16 hours	NH
BADGP; Ferm (1974)	Patients with dementia	Nurse	Not stated	Hospital
BARS; Finkel et al. (1993)	Elderly patients	Nurse	2 weeks	NH
BEAM-D; Sinha et al. (1992)	Patients with dementia	Clinician^***^	Not stated	NH or hospital
BEHAVE-AD; Reisberg et al. (1987)	AD patients	Caregiver	2 weeks	OC
BMDS; Greene et al. (1982)	Elderly patients	Caregiver	Not stated	OC
BPC; O'Malley and Qualls (2016)	Patients with dementia or chronic illness	Caregiver	4–6 months	Home or OC
BPSD-DS; Dekker et al. (2018)	Patients with DS, > 30 years	Clinician^**^	6 months	OC
BPSD-T; Phannarus et al. (2020)	Patients with dementia	Caregiver	1 month	OC
BSO-DOS; BSO-DOS (2019)	Patients with dementia	Nurse	Customizable	Hospital or NH
BSSD; Devanand et al. (1992b)	Patients with dementia	Clinician^*^	1 month	OC
CABOS; Burgio et al. (1994)	Patients with dementia	Nurse	2 weeks	NH
CAMDEX; Roth et al. (1986)	Elderly patients	Clinician^***^	Not stated	OC
CBS; Moniz-Cook et al. (2001)	Patients with dementia	Nurse	8 weeks	NH
CBS-8; Mansbach et al. (2021)	Patients with dementia	Nurse	1 month	NH
CDBQ; Victoroff et al. (1997)	Patients with dementia	Caregiver	1 month (asking approximately in the past 6 months)	OC
CERAD/BRSD; Tariot et al. (1995)	Patients with dementia	Clinician^**^	1 month	OC
CMAI; Cohen-Mansfield et al. (1989b)	Elderly patients	Nurse	Nurse's shift or 2 weeks	NH
COBRA; Drachman et al. (1992)	Patients with dementia	Nurse	Not stated	OC or NH
CPCE; Resnick et al. (2018)	Patients with dementia	Nurse	Not stated	NH
CSDD; Alexopoulos et al. (1988)	Patients with dementia	Clinician^**^	1 week	OC
CUSPAD; Devanand et al. (1992b)	AD patients	Caregiver	1 month	OC
DAIR; Strauss and Sperry (2002)	Patients with dementia	Caregiver	1 month	OC
DBDS; Baumgarten et al. (1990)	Patients with dementia	Caregiver	1 week	OC
DBRI; Molloy et al. (1991)	Elderly patients	Caregiver	Not stated	OC
DBRS; Mungas et al. (1989)	Patients with dementia	Nurse	1 week	NH
DBS; Beck et al. (1997)	Patients with dementia	Nurse	1 week	NH
DMAS; Sunderland et al. (1988)	Patients with dementia	Caregiver	1 week	OC
DSSS; Loreck et al. (1994)	Patients with dementia	Clinician^**^	1 month	OC
HBS; Draper et al. (2002)	Elderly patients	Nurse	2 weeks	NH
IAS; Burns et al. (1990)	AD and HD patients	Clinician^*^	Since the onset of the illness	OC
IQ; Craig et al. (2008)	Psychiatric patients, AD and HD patients	Patient and caregiver	2 weeks	OC
MBI-C; Ismail et al. (2017)	Patients with MBI, > 50 years	Patient, informant, or clinician	6 months	OC
MOUSEPAD; Allen et al. (1997)	Patients with dementia	Caregiver	1 month	OC
NHBPS; Ray et al. (1992)	Patients with dementia	Nurse	3 days	NH
NPI; Cummings et al. (1994)	Patients with dementia	Caregiver	1 month	OC or NH
PAS; Rosen et al. (1994)	Patients with dementia	Nurse	1–8 hours	Hospital or NH
PBE; Hope and Fairburn (1992)	Patients with dementia	Clinician^**^	1 month	OC
PEAR; Jao et al. (2016)	Patients with dementia	Nurse	Not stated	NH
RAGE; Patel and Hope (1992)	Elderly patients	Nurse	3 days	NH
RAID; Shankar et al. (1999)	Patients with dementia	Clinician^**^	2 weeks	OC
RAS; Ryden (1988)	Patients with dementia	Caregiver	Not stated	Home
RMBPC; Teri et al. (1992)	Patients with dementia	Caregiver	Not stated	OC, hospital or NH
RTC-DAT; Mahoney et al. (1999)	Patients with dementia	Nurse	5 min	NH
SDI; Tractenberg et al. (2003)	AD patients	Caregiver	2 weeks	OC
SOAPD; Hurley et al. (1999)	AD patients	Nurse	3 days	NH
SSADE; Takada et al. (2017)	Patients with dementia	Dieticians	1 month	NH
TBS; Asada et al. (1999)	AD patients	Caregiver	Not stated	OC or hospital
WCA; Kales et al. (2017)	Patients with dementia	Caregiver	Not stated	Home

AD, Alzheimer's disease; DS, Down syndrome; HD, Huntington's disease; MBI, Mild behavioral impairment; NH, Nursing home; OC, Outpatient clinic. ^*^Clinician-rated after an interview with informant. ^**^Clinician-rated after an interview with patient and informant. ^***^Clinician-rated after observation and interview with the patient and or informant.

A number of instruments are intended to be used in outpatient memory clinics. Often, the items in these scales have to be rated by caregivers. While the presence of an informant can surely be an advantage compared to the limited significance of behaviors directly observed by the clinician during the relatively short time of an outpatient visit, several general considerations need to be kept in mind. First, the way in which caregivers respond may be influenced by factors related to the scale, such as its length and complexity, and factors related to the caregiver, such as their age, education, gender, relationship with the patient, and even distress associated with this still underrecognized commitment. The complexity of the instrument appears to be the most limiting factor, as emerged from focus groups with caregivers which Kales and colleagues conducted before developing their own tool (Kales et al., 2017). Therefore, it is unlikely that scales like the CDBQ, which has an extremely high number of items, would be suitable for all caregivers (Victoroff et al., 1997). Second, an important issue is caregiver recall bias, which may lead to underreporting or overreporting of psychiatric symptoms in persons living with dementia (La Rue et al., 1992). While the majority of caregiver-rated scales require the informant to recall the last 1–4 weeks, some instruments, such as the BPC or the IAS, ask the rater to recall the last 4–6 months or even from the beginning of the illness, which may not be an optimal strategy for obtaining reliable information (Burns et al., 1990; O'Malley and Qualls, 2016). However, in the case of BPC, it is not clear whether this instrument needs to be used at home, as the authors report that they “elicit caregivers' observations of daily behaviors”. In any case, it is definitely not observational, as caregivers are asked to report their overall judgment about frequency or severity of behaviors.

Another important factor to consider is caregiver burden or distress. While some scales that evaluate this aspect exist and are widely used (Bédard et al., 2001), they are usually intended for any caregiver and are not specifically designed to capture the burden associated with BPSD. Some of the instruments administered to caregivers also include subscales to evaluate BPSD-related distress, which may be useful to identify unmet needs in carers. Examples of these are the BPSD assessment tool—Thai version [BPSD-T (Phannarus et al., 2020)], ABID, BEHAVE-AD, BMDS, BPSD-DS, CDBQ, SDI, and the currently most used version of NPI (Greene et al., 1982; Reisberg et al., 1987; Victoroff et al., 1997; Kaufer et al., 1998; Logsdon et al., 1999; Tractenberg et al., 2003; Dekker et al., 2018). It may be argued that, for most caregivers, separating the severity of a BPSD from its associated burden can be difficult, and collecting data that are expected to be correlated does not provide any added value. However, this may not apply to all cases.

A common way to overcome these limitations in the outpatient clinic is to have the clinician rate the items after a structured or, more rarely, unstructured interview with the patient and/or an informant. In a few cases [such as the ADAS-Noncog and the CAMDEX (Rosen et al., 1984; Roth et al., 1986)], the clinician can base their judgment also on observational data gathered during the visit. A special case is the MBI-C, which can be compiled by the patient, an informant, or the clinician (Ismail et al., 2017).

Instruments intended to be used in NH are almost inevitably rated by nurses and are often based on the direct observation of the patients. The use of (trained) professionals rather than informal caregivers probably increases the ability to discriminate between different items; nevertheless, this is not completely immune from recall bias. While this is minimized when the timing is within a nurse's shift [as in the CMAI, PAS or RTC-DAT (Cohen-Mansfield et al., 1989a; Rosen et al., 1994; Mahoney et al., 1999)], it can still be a problem when the nurse is required to recall periods as long as 2 months [in the Challenging Behaviors Scale, CBS (Moniz-Cook et al., 2001)]. The SSADE, rather than involving nurses, was rated by dietitians involved in nutritional care in NH, with direct observation during mealtimes over the course of a month (Takada et al., 2017).

A few instruments have been adapted for use in hospitals. These include the PAS, the Dementia Observation System (BSO-DOS, 2019), the Behavioral Activities in Demented Geriatric Patients [BADGP (Ferm, 1974)], the Behavioral and Emotional Activities Manifested in Dementia [BEAM-D (Sinha et al., 1992)], the RMBPC (Teri et al., 1992), and the TBS (Asada et al., 1999). Often, these instruments do not state the period of observation, which is probably intended to be tailored to the individual patient. Although considerably more effort has been expended on scales to identify and prevent delirium in geriatric inpatients, BPSD might be quite common in this specific population, with a study finding a prevalence of up to 76% in a general hospital. This may suggest that more useful observational tools need to be developed for assessing BPSD in hospitals, as their presence may result in longer and more expensive stays (Hessler et al., 2018).

Finally, a few instruments can be filled out at home. In the case of RAS or the ABS (Ryden, 1988; Abe et al., 2015), this would be no different from administering the scale in a memory clinic setting. The only tool specifically intended to be used at home is the WeCareAdvisor system (Kales et al., 2017), discussed in Section 3.7.

### 3.5. Considerations of the use of instruments

**Figure 1:**
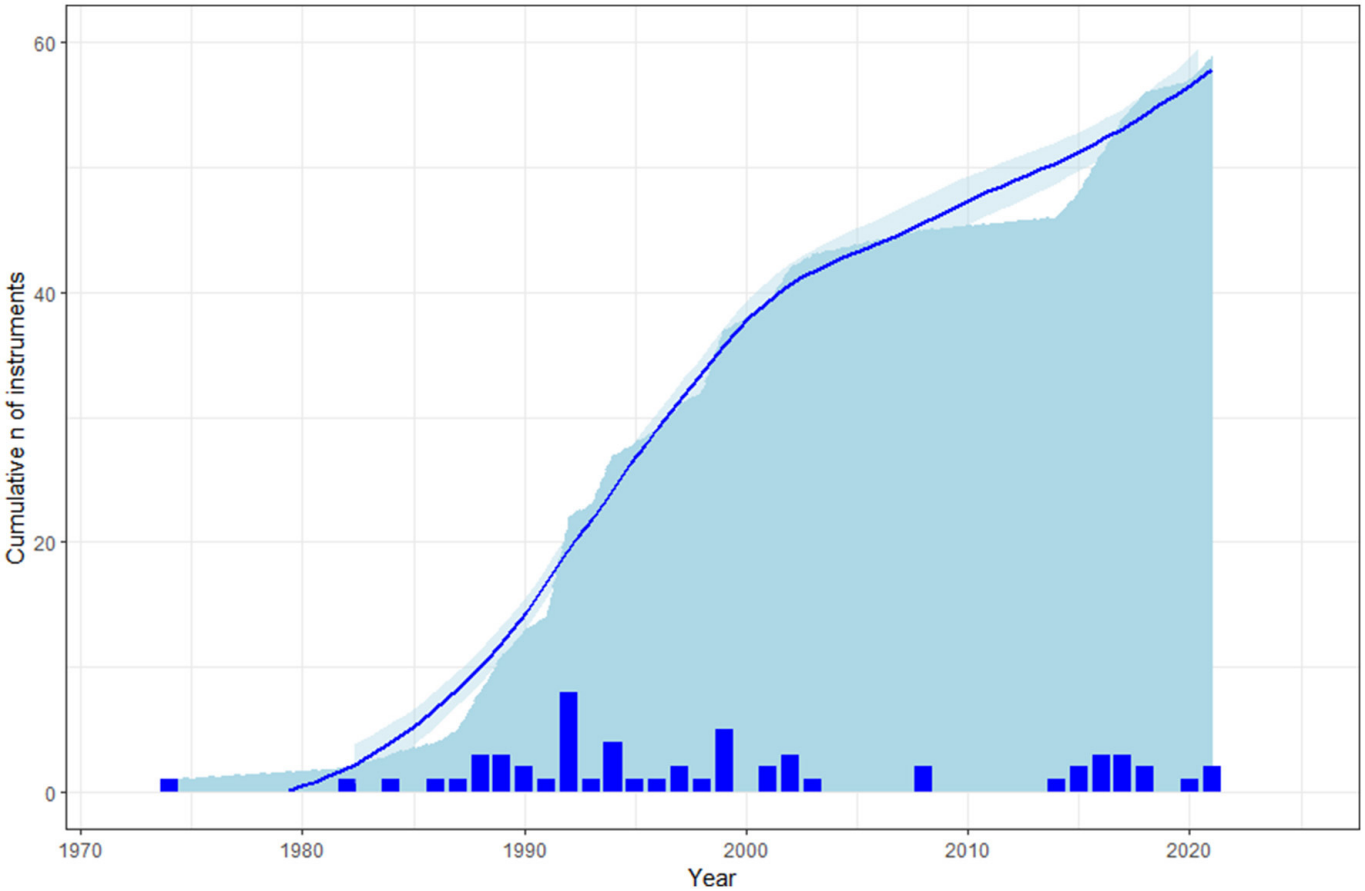
A cumulative graph of the instruments for evaluating BPSD included in the current review by year. The bars represent the number of new instruments for each year.

Although we did not include every possible instrument proposed in the literature, we were still able to define three main phases in the history of BPSD assessment, as shown in the cumulative graph of BPSD tools over time (Figure 1). The first phase, which ran from the 1980s to roughly the year 2000 and saw the proliferation of pen-and-paper scales, includes the vast majority of instruments available today. In the 2000s, only a few new tools were introduced. These were mostly narrow scales, perhaps attempting to fill the gap in the evaluation of specific BPSD domains (this phase corresponds to the plateau in Figure 1). Finally, we owe the steep increase in the number of instruments proposed in the last decade to a combination of scales dedicated to specific issues or populations, on the one hand, and new treatment-oriented approaches, on the other hand.

However, the majority of instruments used nowadays are still those that were introduced in the first phase. For instance, scales such as BEHAVE-AD were used in the trials leading to the approval of risperidone, rivastigmine, and memantine in AD. The BEHAVE-AD underwent two subsequent modifications resulting in the BEHAVE-AD-FW (which adds frequency weights to the already present severity weights) and the E-BEHAVE-AD (an empirical version which includes direct observation of BPSD) (Reisberg et al., 2014).

Table 5 shows various measures estimating the impact of the different tools on research. The most obvious measure is the number of citations of the original study (which was checked with Scopus on 31 December 2022). However, to provide a measure of the centrality of the tool itself, we also report the number of article abstracts that specifically cite it, and the ratio of these mentions to the total number of citations. We normalized citations and mentions in the abstract by the number of years since the tool was introduced. An example of the validity of this approach is shown by the ADAS-Noncog: the original study by Rosen is the second most-cited paper in our list, but it is only mentioned 15 times in the abstract, with considerably less success than the ADAS-Cog (Kueper et al., 2018). This may be due to a number of reasons, including the absence of normative data and the fact that some items are probably not BPSD. The same can be said for the CAMDEX, which is mostly used as an assessment tool for dementia and not primarily for BPSD.

**Table 5:** Usage of different instruments.

**Acronym**	**Year**	**Citations**	**Citations in abstract**	**MA (%)**	**Citations per year**	**MA per year**
NPI; Cummings et al. (1994)	1994	6,042	1,100	18.2	215.0	39.1
ADAS-Noncog; Rosen et al. (1984)	1984	3,481	15	0.4	91.4	0.4
CSDD; Alexopoulos et al. (1988)	1988	2,135	410	19.2	62.6	12.0
CAMDEX; Roth et al. (1986)	1986	1,821	47	2.6	50.4	1.3
BEHAVE-AD; Reisberg et al. (1987)	1987	1118	120	10.7	31.9	3.4
CMAI; Cohen-Mansfield et al. (1989a)	1989	1,045	205	19.6	31.6	6.2
ABMI; Cohen-Mansfield et al. (1989b)	1989	1,031	3	8.6	31.1	0.1
RMBPC; Teri et al. (1992)	1992	898	85	9.5	29.8	2.8
BMDS; Greene et al. (1982)	1982	484	24	5.0	12.1	0.6
CERAD/BRSD; Tariot et al. (1995)	1995	399	28	7.0	14.7	1.0
Apathy Inventory; Robert et al. (2002)	2002	309	33	10.7	15.4	1.6
RAS; Ryden (1988)	1988	251	6	2.4	7.4	0.2
DBDS; Baumgarten et al. (1990)	1990	223	34	15.2	6.9	1.1
CUSPAD; Devanand et al. (1992a)	1992	208	22	10.6	6.9	0.7
MBI-C; Ismail et al. (2017)	2017	186	39	21.0	36.5	7.6
ABeS; Perlman and Hirdes (2008)	2008	163	14	4.4	11.6	1.0
RAID; Shankar et al. (1999)	1999	160	23	14.4	6.9	1.0
IAS; Burns et al. (1990)	1990	151	4	2.6	4.7	0.1
BSSD; Devanand et al. (1992b)	1992	150	0	0	5.0	0
PAS; Rosen et al. (1994)	1994	137	17	12.4	4.9	0.6
DMAS; Sunderland et al. (1988)	1988	136	23	16.9	4.0	0.7
RAGE; Patel and Hope (1992)	1992	135	15	11.1	4.5	0.5
SDI; Tractenberg et al. (2003)	2003	135	13	9.6	7.1	0.7
RTC-DAT; Mahoney et al. (1999)	1999	119	11	9.2	5.2	0.5
NHBPS; Ray et al. (1992)	1992	112	13	11.6	3.7	0.4
CABOS; Burgio et al. (1994)	1994	106	1	0.9	3.8	0
BARS; Finkel et al. (1993)	1993	92	2	2.2	3.2	0.1
DAIR; Strauss and Sperry (2002)	2002	84	8	9.5	4.2	0.4
COBRA; Drachman et al. (1992)	1992	79	1	1.3	2.6	0
AWS; Algase et al. (2001)	2001	73	6	8.2	3.5	0.3
MOUSEPAD; Allen et al. (1997)	1996	73	5	6.8	2.8	0.2
PBE; Hope and Fairburn (1992)	1992	73	16	21.9	2.4	0.5
ABID; Logsdon et al. (1999)	1999	68	3	0.3	2.9	0.1
DBRS; Mungas et al. (1989)	1989	64	0	0	1.9	0
IQ; Craig et al. (2008)	2008	57	7	12.3	4.0	0.5
BADGP; Ferm (1974)	1974	47	1	2.1	1.0	0
SOAPD; Hurley et al. (1999)	1999	47	2	4.3	2.0	0.1
BEAM-D; Sinha et al. (1992)	1992	44	2	4.5	1.5	0.1
HBS; Draper et al. (2002)	2002	41	4	9.8	2.0	0.2
TBS; Asada et al. (1999)	1999	39	12	30.8	1.7	0.5
CBS; Moniz-Cook et al. (2001)	2001	38	5	13.2	1.8	0.2
ABS; Abe et al. (2015)	2015	37	4	10.8	5.2	0.6
DBRI; Molloy et al. (1991)	1991	37	3	8.1	1.2	0.1
BPSD-DS; Dekker et al. (2018)	2018	32	3	9.4	7.8	0.7
DSSS; Loreck et al. (1994)	1994	29	1	3.4	1.0	0
DBS; Beck et al. (1997)	1997	23	1	4.3	0.9	0
WCA; Kales et al. (2017)	2016	22	0	0	3.6	0
PEAR; Jao et al. (2016)	2016	18	6	33.3	3.0	1.0
ACID; Gerolimatos et al. (2015)	2015	14	0	0	2.0	0
CPCE; Resnick et al. (2018)	2017	10	1	10	2.0	0.2
CDBQ; Victoroff et al. (1997)	1997	9	0	0	0.4	0
ABC-DS; Kikuchi et al. (2018)	2018	7	3	42.9	1.7	0.7
SSADE; Takada et al. (2017)	2017	4	0	0	0.8	0
BPC; O'Malley and Qualls (2016)	2016	3	0	0	0.5	0
BPSD-T; Phannarus et al. (2020)	2020	2	0	0	1.0	0
CBS-8; Mansbach et al. (2021)	2021	0	0	0	0	0
BSO-DOS; BSO-DOS (2019)	1998	No data	No data	-	-	-

MA, Instrument mentioned in abstract.

Based on these data, it appears that the NPI is by far the most widely used instrument in clinical studies nowadays, and several additional versions have been developed, such as the NPI-12 [with the addition of eating and sleeping problems, which is now the standard (Cummings, 1997), together with the caregiver distress scale (Kaufer et al., 1998)], the NPI-Q [which is a quick tool administered to the caregiver (Kaufer et al., 2000)], the NPI-nursing home [NPI-NH (Wood et al., 2000), for which a diary version, the NPI-Diary, has also been developed (Morganti et al., 2018)], the NPI-plus [including a 13th item, cognitive fluctuations, and intended for patients with dementia with Lewy bodies (Kanemoto et al., 2021)], the NPI-C [an expanded clinician-rated version (De Medeiros et al., 2010)], and a mobile version developed in Thailand (Rangseekajee et al., 2021).

A few other scales have been subject to further modifications in a similar way to expand and adapt them to other settings. For instance, revisions to the AWS have resulted in a second version with more items [AWS2 (Algase et al., 2004a)] and in a version for individuals living in the community to be compiled by the caregiver [AWS-CV (Algase et al., 2004b)]. The BPSD-DS also underwent a revision and is now available in a second version [BPSD-DS II (Dekker et al., 2021)]. The CAMDEX was also revised [CAMDEX-R (Roth et al., 1998; Ball et al., 2004a)] and adapted for persons with Down syndrome [CAMDEX-DS and CAMDEX-DS II (Ball et al., 2004a)]. The MOUSEPAD also exists in a shorter version [mini-MOUSEPAD (Ball et al., 2004b)] and so do the DBDS [DBD13 (Machida, 2012)], the CERAD/BRSD [with a 17-item version (Fillenbaum et al., 2008)], and the CSDD [mCSDD4-MA, a four-item scale for advanced dementia (Niculescu et al., 2021)].

The BEHAVE-AD, CSDD, and CMAI are other widely used scales, and it is possible, based on the available data, that newer instruments such as the MBI-C may achieve comparable success in the near future.

Another factor that may be both a cause and an effect of an instrument's success is language availability. Table 6 lists the languages in which the various instruments have been used in the literature, with a focus on the 10 currently most spoken languages (https://www.ethnologue.com/guides/ethnologue200). However, we excluded Bengali, Russian, and Urdu from the columns of the table, as we are not aware of any scale other than the NPI being translated into these languages. The high number of languages into which they have been translated testifies to the success of instruments such as the NPI, CSDD and BEHAVE-AD. Regrettably, almost half of the tools are only available in English. Another way of looking at Table 6 is as a rough estimate of the scientific interest in BPSD assessment of different communities, which is not always proportional to their size. While it is foreseeable that most scales have been adapted for Spanish, French, Chinese or even Japanese, the fact that many of them have been validated in Italian (10 scales), Dutch (7), Korean (7), and Norwegian (5) probably reflects the great involvement of the respective countries in BPSD research.

**Table 6:** Languages in which each instrument has been used or is available.

**Acronym**	**English**	**Chinese**	**Hindi**	**Spanish**	**French**	**Arabic**	**Portuguese**	**Other languages**
ABC-DS; Kikuchi et al. (2018)	Yes	Yes	-	-	Yes	-	-	Korean
ABID; Logsdon et al. (1999)	Yes	-	-	-	-	-	-	Japanese
ABMI; Cohen-Mansfield et al. (1989a)	Yes	-	-	-	-	-	-	-
ABeS; Perlman and Hirdes (2008)	Yes	Yes	-	-	-	-	-	-
ABS; Abe et al. (2015)	Yes	-	-	-	-	-	Yes	Japanese
ACID; Gerolimatos et al. (2015)	Yes	-	-	-	-	-	-	-
ADAS-Noncog; Rosen et al. (1984)	Yes	-	-	Yes	Yes	-	-	Italian, Swedish, and German
Apathy Inventory; Robert et al. (2002)	Yes	-	-	Yes	Yes	-	Yes	Korean
AWS; Algase et al. (2001)	Yes	-	-	-	Yes	-	-	German, Korean, and Japanese
BADGP; Ferm (1974)	Yes	-	-	-	-	-	-	Finnish
BARS; Finkel et al. (1993)	Yes	-	-	-	-	-	-	-
BEAM-D; Sinha et al. (1992)	Yes	-	-	-	-	-	-	-
BEHAVE-AD; Reisberg et al. (1987)	Yes	Yes	-	Yes	Yes	-	-	Dutch, Japanese, German, Italian, and Korean, Cantonese
BMDS; Greene et al. (1982)	Yes	Yes	-	-	-	-	-	Italian and Norwegian
BPC; O'Malley and Qualls (2016)	Yes	-	-	-	-	-	-	-
BPSD-DS; Dekker et al. (2018)	Yes	-	-	Yes	Yes	-	-	Italian and Dutch
BPSD-T; Phannarus et al. (2020)	Yes	-	-	-	-	-	-	Thai
BSO-DOS; BSO-DOS (2019)	Yes	-	-	-	Yes	-	-	-
BSSD; Devanand et al. (1992a)	Yes	-	-	-	-	-	-	-
CABOS; Burgio et al. (1994)	Yes	-	-	-	-	-	-	-
CAMDEX; Roth et al. (1986)	Yes	-	-	Yes	-	-	Yes	German, Dutch, Italian, Polish, and Danish
CBS; Moniz-Cook et al. (2001)	Yes	Yes	-	-	-	-	-	Hebrew
CBS-8; Mansbach et al. (2021)	Yes	-	-	-	-	-	-	-
CDBQ; Victoroff et al. (1997)	Yes	-	-	-	-	-	-	-
CERAD/BRSD; Tariot et al. (1995)	Yes	-	-	-	Yes	-	-	Korean
CMAI; Cohen-Mansfield et al. (1989b)	Yes	Yes	-	Yes	Yes	-	-	Italian, Dutch, German, Slovak, and Turkish, Norwegian, Japanese, Polish, and Korean
COBRA; Drachman et al. (1992)	Yes	-	-	-	-	-	-	-
CPCE; Resnick et al. (2018)	Yes	-	-	-	-	-	-	-
CSDD; Alexopoulos et al. (1988)	Yes	Yes	-	Yes	Yes	Yes	Yes	Norwegian, Vietnamese, Italian, Dutch, and Persian, Japanese, Korean, Turkish, and Finnish, Thai, Malaysian, German, and Danish
CUSPAD; Devanand et al. (1992b)	Yes	-	-	-	-	-	-	German and Greek
DAIR; Strauss and Sperry (2002)	Yes	-	-	Yes	-	-	-	-
DBDS; Baumgarten et al. (1990)	Yes	Yes	-	Yes	-	-	-	Japanese and Serbian
DBRI; Molloy et al. (1991)	Yes	-	-	-	-	-	-	-
DBRS; Mungas et al. (1989)	Yes	-	-	-	-	-	-	-
DBS; Beck et al. (1997)	Yes	-	-	-	-	-	-	-
DMAS; Sunderland et al. (1988)	Yes	-	-	-	-	-	-	Japanese and German
DSSS; Loreck et al. (1994)	Yes	-	-	-	-	-	-	-
HBS; Draper et al. (2002)	Yes	-	-	-	-	-	-	-
IAS; Burns et al. (1990)	Yes	-	-	-	-	-	-	-
IQ; Craig et al. (2008)	Yes	-	-	-	-	-	-	Persian
MBI-C; Ismail et al. (2017)	Yes	Yes	-	Yes	Yes	-	-	Italian, Persian, and Czech
MOUSEPAD; Allen et al. (1997)	Yes	-	-	-	-	-	-	-
NHBPS; Ray et al. (1992)	Yes	-	-	-	Yes	-	-	Danish
NPI; Cummings et al. (1994)	Yes	Yes	Yes	Yes	Yes	-	Yes	Icelandic, Italian, Danish, Polish, and Thai, Japanese, Hebrew, German, Czech, and Korean, Dutch, Finnish, Turkish, and Serbian, Norwegian, Swedish, Greek, Croatian, and Russian, Bengali, and Urdu (only NPI-10)
PAS; Rosen et al. (1994)	Yes	Yes	-	Yes	-	-	-	Persian
PBE; Hope and Fairburn (1992)	Yes	-	-	-	-	-	-	-
PEAR; Jao et al. (2016)	Yes	-	-	-	-	-	-	-
RAGE; Patel and Hope (1992)	Yes	Yes	-	Yes	Yes	-	-	Japanese
RAID; Shankar et al. (1999)	Yes	Yes	-	Yes	-	Yes	-	Persian and Norwegian
RAS; Ryden (1988)	Yes	-	-	-	-	-	-	-
RMBPC; Teri et al. (1992)	Yes	Yes	-	Yes	-	-	-	Italian, German, Thai, and Dutch
RTC-DAT; Mahoney et al. (1999)	Yes	-	-	-	-	-	-	Swedish
SDI; Tractenberg et al. (2003)	Yes	-	-	Yes	Yes	-	-	Japanese, German, Korean, and Norwegian
SOAPD; Hurley et al. (1999)	Yes	-	-	-	-	-	-	Korean
SSADE; Takada et al. (2017)	Yes	-	-	-	-	-	-	Japanese
TBS; Asada et al. (1999)	Yes	-	-	-	-	-	-	Japanese
WCA; Kales et al. (2017)	Yes	-	-	-	-	-	-	-

Cross-cultural considerations need to be taken into account, as some instruments are specifically designed for issues that might not be as relevant in a different context. For instance, a number of scales developed in Japan include items related to toileting behaviors (Asada et al., 1999; Abe et al., 2015; Mori et al., 2018) or to aspects that seem less easy to rate objectively, such as interfering with a happy home circle (Asada et al., 1999). These BPSD are rarely mentioned in Western scales (incontinence is more often listed as a physical issue, rather than a behavior). Conversely, disinhibition, anxiety, and shadowing are almost never present in Japanese instruments. Although it is possible that some variation exists in the geographic prevalence of specific BPSD, it cannot be excluded that some behaviors, albeit present, are not perceived as issues in different cultures. Therefore, it is advisable to use instruments that are relevant to the context in which they are applied.

One important factor regarding usability is the way an instrument is scored and how the scale is interpreted. A significant number of instruments do not provide score cutoffs or interpretations, leaving the clinician with a tool that they might not know how to interpret. Even in longitudinal retest after treatment, it is not always clear how to consider changes, and the real additive value of even using a score is questionable if the overall judgment still has to be done in a rather personal way. This is especially true for broad instruments. To give a practical example of this issue, let us consider two different patients with an NPI of 24 points, the first resulting from 12 on apathy and depression, the second from 12 on aggression and delusions. After treatment, the first one obtains a score of 18 (with nine on apathy and depression), while the second gets a score of 12 (with no more delusions, but still 12 on aggression). It would appear that the second patient has improved more than the first, but, conversely, one might also judge that a 6-point change in the first case could be enough, while a 12-point change in the second case would still be far from acceptable.

Table 7 provides an overview of the scoring rules, the modalities that are evaluated, and the subscales of each instrument. With a few exceptions, the vast majority of the tools are based on Likert scales that evaluate frequency, severity, or both, and, in some cases, also caregiver distress. Alternatives include averaging subscale scores (also based on Likert scales) to make a total score and, in a few cases, using weighted coefficients for each item. These approaches make the use of the instrument more complex, as they imply calculations beyond simple sums [or sum of products, as in the case of the NPI, CBS, SDI, or the Apathy Inventory (Cummings et al., 1994; Moniz-Cook et al., 2001; Robert et al., 2002; Tractenberg et al., 2003)]. An extreme example of this has been provided by the ABC-DS, for which the authors proposed to calculate the Euclidean tridimensional distance between domains as a measure of the total score, with the additional caveat that each item is rated in an unusually decreasing way, with higher scores corresponding to less impairment (Kikuchi et al., 2018). A few tools do not provide any score at all, but rather a treatment-oriented analysis of the impact of behaviors, often in conjunction with an evaluation of possible triggers; these are discussed in the following section. Table 7 also provides a personal indication of the usability of each tool on a qualitative scale in the “Ease of use” column, where “-” means that the instrument is probably too complex, and “+++” means that it is extremely easy, mostly based on its length and scoring rules.

**Table 7:** Scoring rules and usability of the scales.

**Acronym**	**Score rules**	**TS**	**Modalities**	**Range F**	**Range S**	**Range D**	**Other subscales**	**Range OS**	**Trigger**	**EoU**
ABC-DS; Kikuchi et al. (2018)	Distance among domains	70.29–7.81	-	-	-	-	ADL, BPSD, Cognition	27-3 (domain B)	-	+
ABID; Logsdon et al. (1999)	LS	-	F, D	0-96	-	0-64	-	-	-	++
ABMI; Cohen-Mansfield et al. (1989a)	Number of behaviors	0–140	F	0-140	-	-	Disruptiveness	Not al all - extreme	Yes	++
ABeS; Perlman and Hirdes (2008)	LS	0–12	F	0-12	-	-	-	-	-	+++
ABS; Abe et al. (2015)	Score sheet	0-44	F	0-44	-	-	-	-	-	++
ACID; Gerolimatos et al. (2015)	LS	-	Presence and D	-	-	-	Self-report and proxy-report	0-26 (each)	-	++
ADAS-Noncog; Rosen et al. (1984)	LS	0-50	S	-	0–50	-	-	-	-	+
Apathy Inventory; Robert et al. (2002)	LS	0–36	F x S	-	-	-	-	-	-	++
AWS; Algase et al. (2001)	LS	29–116	F or S	-	-	-	5 dimensions	Not clear	-	++
BADGP; Ferm (1974)	LS	13–78	F	-	-	-	-	-	-	+++
BARS; Finkel et al. (1993)	LS	10–70	F	10–70	-	-	-	-	-	+++
BEAM-D; Sinha et al. (1992)	LS	0–69	S	0–69	-	-	-	-	-	+++
BEHAVE-AD; Reisberg et al. (1987)	LS	-	S, D	-	0–75	0–3	A-G	Variable	-	+
BMDS; Greene et al. (1982)	LS	-	F or S, D	0–136^*^	0–136^*^	0–60	-	-	-	++
BPC; O'Malley and Qualls (2016)	ALS	Not clear	F	Not clear	-	-	BPSD, ADL, and Attributions	Not clear	-	++
BPSD-DS; Dekker et al. (2018)	Subtraction of LS	-	F and S	−48 to +48	−36 to +36	0–36	-	-	-	++
BPSD-T; Phannarus et al. (2020)	LS	-	F, D	14–56	-	14–56	-	-	-	+++
BSO-DOS; BSO-DOS (2019)	Analysis table	-	F	-	-	-	-	-	Yes	++
BSSD; Devanand et al. (1992a)	LS	-	S	-	Not clear	-	6 subscales	Not clear	-	+
CABOS; Burgio et al. (1994)	Descriptive statistics	-	F	-	-	-	-	-	Yes	+
CAMDEX; Roth et al. (1986)	Variable	-	-	-	-	-	A-H (BPSD in A and H)	Not clear	-	+
CBS; Moniz-Cook et al. (2001)	LS	0–400	F x S	0–100	0–100	-	-	-	-	++
CBS-8; Mansbach et al. (2021)	LS	0–24	F	0–24	-	-	-	-	-	+++
CDBQ; Victoroff et al. (1997)	LS	-	F, S, D	62–248	19-57	Not clear (6–24?)	Depression, psychosis, and agitation	28–123 (D), 19–91 (P), 20–85 (A)	-	++
CERAD/BRSD; Tariot et al. (1995)	LS	Not clear	F, validity of rating	Not clear	-	-	-	-	-	+
CMAI; Cohen-Mansfield et al. (1989b)	LS	29–203	F	29-203	-	-	-	-	-	++
COBRA; Drachman et al. (1992)	LS	-	F, S	Not clear	Not clear	-	12 different summary scores	Not clear	-	+
CPCE; Resnick et al. (2018)	Checklist	0–8	Presence, addressing of behavior	-	-	-	-	-	-	+++
CSDD; Alexopoulos et al. (1988)	LS	0–38	F and S	0–38^*^	0–38^*^	-	-	-	-	+++
CUSPAD; Devanand et al. (1992b)	Score sheet	Not clear	Presence or features	-	-	-	-	-	-	+
DAIR; Strauss and Sperry (2002)	ALS	0–3	F	0–3	-	-	-	-	-	+++
DBDS; Baumgarten et al. (1990)	LS	0–112	F	0–112	-	-	-	-	-	+++
DBRI; Molloy et al. (1991)	LS	0–125	F	0–125	-	-	-	-	-	+++
DBRS; Mungas et al. (1989)	ALS	0–5	S	-	0–5	-	4 subscales	0–5 (each)	-	++
DBS; Beck et al. (1997)	WC	Not clear	F and S	-	-	-	-	-	-	-
DMAS; Sunderland et al. (1988)	LS	Not clear	S	-	Not clear	-	-	-	-	+
DSSS; Loreck et al. (1994)	LS	Not clear	F and/or S	Not clear	Not clear	-	5 subscales	Variable	-	-
HBS; Draper et al. (2002)	LS	0-92	F	0-92	-	-	-	-	-	+++
IAS; Burns et al. (1990)	LS	-	F and/or S	-	-	-	Irritability, apathy	5–17 (I), 5–25 (A)	-	++
IQ; Craig et al. (2008)	LS	-	F, S	0–63	0–63	-	Caregiver (F & S)	0–30 (each)	-	++
MBI-C; Ismail et al. (2017)	LS	0-102	S	-	0–102	-	-	-	-	++
MOUSEPAD; Allen et al. (1997)	LS	Not clear	F and S	Not clear	Not clear	-	-	-	-	+
NHBPS; Ray et al. (1992)	LS	0-116	F	0-116	-	-	-	-	-	+++
NPI; Cummings et al. (1994)	LS	0-120	F x S	-	-	0–50	-	-	-	++
PAS; Rosen et al. (1994)	Score sheet	0-16	S	-	0–16	-	-	-	-	+++
PBE; Hope and Fairburn (1992)	Variable	Not clear	Not clear	-	-	-	-	-	-	-
PEAR; Jao et al. (2016)	Score sheet	6-24	S	-	6–24	-	Environment	6–24	-	++
RAGE; Patel and Hope (1992)	LS	0-61	F and/or S	-	-	-	-	-	-	++
RAID; Shankar et al. (1999)	LS	0–54	S	-	0-54	-	-	-	-	+++
RAS; Ryden (1988)	LS	0–125	F	0-125	-	-	Physical, verbal, and sexual aggression	0-80 (P), 0-20 (V), 0-25 (S)	-	+++
RMBPC; Teri et al. (1992)	LS	0–96	F, D	0-96	-	0-96	-	-	-	++
RTC-DAT; Mahoney et al. (1999)	LS	0–156	Duration x intensity	-	-	-	-	-	-	++
SDI; Tractenberg et al. (2003)	ALS	0–12	F x S	-	-	0-5	-	-	-	+
SOAPD; Hurley et al. (1999)	WC	0–4445.3	Duration, intensity	-	-	-	-	-	-	-
SSADE; Takada et al. (2017)	LS	-	F, S	11–55	11–55	-	-	-	-	+++
TBS; Asada et al. (1999)	LS	0-56	F	0–56	-	-	-	-	-	+++
WCA; Kales et al. (2017)	Checklist	-	Features	-	-	-	-	-	Yes	++

ALS, Average of Likert scales; D, caregiver distress; EoU, ease of use; F, frequency; LS, Likert scales; WC, weighted coefficients; S, severity; TS, total score. Abbreviations in the “Range OS (Range other subscales)” are the initials of the subscales on the left. Asterisks in the “Range F” and “Range S” columns mean that they are evaluated with a single score; absence of asterisks implies that two separate scales exist.

Another important consideration is the availability of the instrument. While the vast majority of published tools are free and—at least for the more recent ones—often included in open access papers, some others are intended for commercial use, such as the CAMDEX (Roth et al., 1986), the ABC-DS (Kikuchi et al., 2018), or the WeCareAdvisor platform (Kales et al., 2017). While the costs are not prohibitive in most cases, ranging from USD$ 20–130, the fact that they still need to be paid would probably make them less likely to be used worldwide.

### 3.6. Internal consistency and reliability of the instruments

The scales and instruments described above are often used as outcomes in clinical trials aiming at establishing the efficacy of interventions in BPSD. Therefore, it is crucial to be aware of their reliability, as this may influence the statistical power of these trials (Matheson, 2019). In this context, reliability can be understood as the ratio of the variance of the true score of a scale (without random errors) to the variance of the total score (which includes random errors) (David, 2003) or as the proportion of the variability of the score that is attributable to what the score should measure in relation to the total variability of the person's responses (Dunn et al., 2014).

However, as the reliability of a measure is dependent on its calibration within a specific sample and in a specific context (Dunn et al., 2014), the data provided by the original papers cannot always be assumed to reflect the real-world reliability of the respective instrument, as the samples to which that scale is to be applied might be more or less heterogeneous than the original sample (David, 2003). In other words, an instrument that was judged reliable enough within a sample of relatively young Canadian caregivers might not be reliable in a sample of older Italian caregivers. Keeping this in mind, in this section we have provided an overview of the reliability measurements of the different instruments as measured in the original papers. Scales for which reliability data are available are shown in Table 8. Moreover, education of the caregiver, although important, was left out, as it was available only for the ABID (13.3 ± 2.6 years).

**Table 8:** Reliability measures of the different scales. Only scales for which reliability data are available are shown.

**Acronym**	**Sample (P)**	**Age (P)**	**Education (P)**	**Age (C)**	**Cronbach alpha**	**IRR (ICC)**	**TRT (ICC)**	**Type**
ABC-DS; Kikuchi et al. (2018)	312 AD	-	-	-	-	-	0.964	N
ABID; Logsdon et al. (1999)	148 AD	74.8 ± 7.1	12.6 ± 3.5	65.6 ± 13.0	0.70	-	0.73 (frequency), 0.60 (reaction)	B
ABeS; Perlman and Hirdes (2008)	214 PwD	82.0 ± 11.3	-	-	0.80	-	-	N
ACID; Gerolimatos et al. (2015)	45 PwD	77.6 ± 11.0	11.9 ± 3.9	-	0.73–0,87^*^	-	-	B
ADAS-Noncog; Rosen et al. (1984)	27 AD	65.1 ± 7.4	14.6 ± 2.6	-	–	0.95 (original)	-	B
Apathy Inventory; Robert et al. (2002)	115 AD	74.9 ± 7.1	-	-	0.84 (caregiver)	-	-	N
AWS; Algase et al. (2001)	151 PwD	85.7 ± 6.5	-	-	0.86	-	-	N
BARS; Finkel et al. (1993)	232 EP	86 [65–102]	-	-	0.74–0.82^**^	-	-	N
BPC; O'Malley and Qualls (2016)	456 PwD/CI	79 ± 11.6	-	60 ± 11.1	0.88–0.93^*^	-	-	B
BPSD-DS; Dekker et al. (2018)	? DS	-	-	-	0.88–0.90^*^	-	-	B
BPSD-T; Phannarus et al. (2020)	168 PwD	80.7 ± 6.7	-	-		-	-	B
BSSD; Devanand et al. (1992a)	106 PwD	72.1 ± 9.8	11.5 ± 4.2	-	0.69–0.83^*^	0.76–0.95^*^	-	B
CBS; Moniz-Cook et al. (2001)	382 PwD	82.7 ± 8.1	-	-	0.82–0.87^*^	0.59−0.66^*^	0.97–0.99	B
CBS-8; Mansbach et al. (2021)	350 PwD	79.1 ± 10.2	-	-	0.78	1	-	B
CDBQ; Victoroff et al. (1997)	258 PwD	-	-	-	0.91–0.92^*^	-	-	B
CPCE; Resnick et al. (2018)	137 PwD	82.0 ± 11.4	-	-	0.96	-	-	B
CSDD; Alexopoulos et al. (1988)	83 PwD	79 [63–92]	-	-	0.84	-	-	B
CUSPAD; Devanand et al. (1992a)	91 AD	72.0 ± 9.8	-	-	-	-	-	B
DAIR; Strauss and Sperry (2002)	100 PwD	75.0 ± 8.5	-	-	0.89	-	-	N
DBDS; Baumgarten et al. (1990)	96 PwD	77.8 ± 6.2 (sample 1), 68.9 ± 8.2 (sample 2)	-	-	0.83–0.84	-	-	B
DBRI; Molloy et al. (1991)	184 EP	-	-	-	-	-	0.75	B
DBRS; Mungas et al. (1989)	16 PwD	-	-	-	-	0.90	-	N
DBS; Beck et al. (1997)	62 PwD	72.9 ± 5.5	-	-	-	0.80	-	B
DMAS; Sunderland et al. (1988)	21 PwD	66.9 ± 10.2	-	-	-	0.69–0.74	-	B
DSSS; Loreck et al. (1994)	56 PwD	73.2 ± 7.6	12.3 ± 4.1	-	0,37–0,75	0.92–0.99	-	B
HBS; Draper et al. (2002)	610 EP	83.9 [65-111]	-	-	0,85–0,87^**^	0.90 [0.81–0.94]	-	B
IAS; Burns et al. (1990)	31 (AD), 26 (HD)	70.3 ± 7.3 (AD), 48.3 ± 15.1 (HD)	-	-	0.78–0.82^*^	-	-	N
IQ; Craig et al. (2008)	64 (AD, HD, PP)	70 ± 8 (AD), 51 ± 9 (HD)	-	-	0.90	-	-	N
NPI; Cummings et al. (1994)	40 PwD	75.7 [56–90]	-	-	0.88	-	-	B
PAS; Rosen et al. (1994)	25 PwD	-	-	-	-	0.82 (hospital), 0.93 (NH)	-	N
PEAR; Jao et al. (2016)	24 PwD	82.4 ± 7.0	-	-	0.84–0.85^*^	-	-	N
RAGE; Patel and Hope (1992)	? EP	-	-	-	0.89	-	-	B
RAID; Shankar et al. (1999)	83 PwD	79.1 ± 7.0	-	-	0.83	-	-	N
RAS; Ryden (1988)	183 PwD	71.1 [45-87]	-	-	0.74–0.90^*^	-	-	N
RMBPC; Teri et al. (1992)	201 PwD	74 ± 8.1	-	54 ± 13.1	0.67–0.90^*^	-	-	B
RTC-DAT; Mahoney et al. (1999)	68 PwD	72.8 ± 7.7	12.2 ± 3.8	-	0.87	-	-	N
SOAPD; Hurley et al. (1999)	57 AD	82.7 ± 7.5	-	-	0.70	-	-	N
SSADE; Takada et al. (2017)	258 PwD	85.7 ± 7.2	-	-	0.24–0.91 (items)	-	-	N

Data are shown with means ± SD; or mean [range]. ^*^Statistics that refer to subscores or subscales; rather than to the total score. ^**^Statistics that refer to different shifts. B, broad instruments; C, caregivers; CI, chronic illness patients; DS, Down syndrome; EP, elderly patients; HD, Huntington's disease; IRR, inter-rater reliability; N, narrow instruments; P, patients; PP, psychiatric patients; PwD, patients with dementia; TRT, test-retest reliability.

Reliability can usually take one of the three forms: internal consistency, inter-rater reliability, and test-retest reliability. Internal consistency is generally understood as the ratio of the variability between the responses to individual items of a scale and the overall variability of the scale. It is generally measured with Cronbach's alpha, although several limitations and interpretation caveats should be borne in mind with this approach. First, Cronbach's alpha depends on how much the individual items correlate between themselves, and as such, it is expected to be lower for broad constructs and higher for narrow ones. Moreover, while a very low (or even negative) alpha would reflect general disagreement between the items, a very high coefficient would be indicative of redundancy (in this sense, an alpha higher than 0.90 should raise suspicion). Indeed, high alphas seem to be artificially driven in many scales by the use of indefensibly similar items and/or multiplication of items describing the same latent variable, as alpha is also proportional to the number of items (David, 2003). As shown more than 30 years ago, a scale with poor average item correlation (0.30) and three independent dimensions will have an alpha of 0.28 with six items, and an alpha of 0.64 with 18 items (Cortina, 1993). For instance, a scale with 10 items for apathy and 4 items for depression will predictably show a higher alpha than a scale with just two items for each category, but the latter would probably be less redundant and time-consuming. Yet, over the past three decades, a number of scales with an enormously large number of items have been published, with Cronbach's alphas provided in an attempt to justify the value of the instrument.

Another common misconception is that the adaptation of original scales on the basis of alpha after removal of items would result in a better scale. This is a common methodological error, as even in the context of unidimensionality of the scale, the item removed might have lower variance than other items and, therefore, be more reflective of a “true” value within the sample (Dunn et al., 2014).

Furthermore, alpha is often inappropriately reported as a point estimate (Dunn et al., 2014), and no article gives confidence intervals for alphas. However, one has to bear in mind that the smaller the sample, the more imprecise the estimate of alpha (Knapp, 1991). Another common poor practice in articles is to provide total Cronbach's alphas (and total scores for the instrument) even when factor analyses demonstrate that there are many poorly correlated dimensions evaluated by the instrument (Knapp, 1991). This is especially applicable to broad instruments, where a total score is usually a poor indication of the real problems of the patient. However, even as an index of unidimensionality, Cronbach's alpha performs relatively poorly compared to other less-used indexes, such as McDonald's omega (Revelle and Zinbarg, 2009; Dunn et al., 2014). Finally, there are several different alphas, such as Cronbach's alpha, standardized alpha (which is actually not an alpha), and weighted alpha, which is more suitable for scales where different items contribute in different ways to the total score (Knapp, 1991). Therefore, interpretation of the values provided is not always straightforward. The data in Table 8 will help the reader put the given alphas into context.

Inter-rater reliability measures the variation between two or more raters evaluating the same sample, while test-retest reliability measures the consistency of the results when the same test is repeated at different time points within the same sample. Both can be estimated with an intraclass correlation coefficient (ICC), but ICC does not represent a single measure, as there are up to 10 ways to calculate it, each of which is subject to different assumptions (model, actual measurement protocol and interest in absolute agreement vs consistency between raters) and interpretations (Koo and Li, 2016). Although the two-way random-effect model with absolute agreement would be the preferred ICC for inter-rater reliability because of its generalizability, and the two-way mixed-effect model should be used for test-retest reliability studies (Koo and Li, 2016), the specification of the ICC is rarely reported. As for Cronbach's alpha, ICCs are dependent on the degree of homogeneity of the sample for which they are calculated, and therefore, caution should be applied when extending its value to other populations (Aldridge et al., 2017; Matheson, 2019). Moreover, crude intra-observer agreement, Pearson's r, and Cohen's k are often used, even though Pearson's r represents only a correlation coefficient (but, on top of its several limitations, correlation does not imply agreement) (Aldridge et al., 2017), and Cohen's k is intended as a measure of inter-rater agreement for nominal data (Cohen, 1960), while most scales are actually based on ordinal or interval data. For ordinal rating scales, weighted Cohen's k is more appropriate (Cohen, 1968; de Raadt et al., 2021), but the way in which disagreement between observers can be regarded varies somewhat arbitrarily, depending on the weight model, as quadratic weights consider distant ratings more seriously than linear weights (de Raadt et al., 2021). However, as for the ICC, the type of weight used is not often reported, and the arbitrariness of the decision on the specific weights has led some authors to advise against its use (de Raadt et al., 2021). For the sake of consistency, only values for ICC are reported in Table 8. Traditionally, values between 0.4 and 0.6 have been considered fair, between 0.6 and 0.74 good, and above 0.75 excellent (Matheson, 2019).

For test-retest reliability, it is also important to know the time frame and the sample for which it has been estimated. Samples for test–reliability range from 35 days of the DBRI to 218 days of the ABC-DS, while the time frames are generally between one and two weeks.

Finally, concurrent validity is regarded as a measure of correlation between a new scale or instrument and a validated gold standard. Such a reference scale is obviously dependent on the type of tool to be assessed (broad vs narrow) and on historical considerations. For broad instruments, the gold standard is probably the NPI, but concurrent validity can only be established for scales published after its introduction. For narrow instruments, a series of scales have been used as comparators, depending on their status at the time of publication. Table 9 lists the concurrent validity of each instrument as provided in the original paper.

**Table 9:** Correlations between instruments.

**Acronym**	**NPI correlation (r)**	**Type of NPI**	**Other correlations (r)**
ABC-DS; Kikuchi et al. (2018)	−0.64	NPI-distress	-
ABID; Logsdon et al. (1999)	-	-	RMBPC (0.74), CERAD/BRSD (0.65), CMAI (0.62)
ABS; Abe et al. (2015)	0.72	NPI	-
ABeS; Perlman and Hirdes, 2008)	-	-	CMAI–aggression subscore (0.72)
ACID; Gerolimatos et al. (2015)	0.27 (SR) - 0.52 (PR)	NPI-anxiety	CSDD-Person (0.71, SR), CSDD-Proxy (0.81, PR)
Apathy Inventory; Robert et al. (2002)	Not significant	NPI-disphoria	-
BARS; Finkel et al. (1993)	-	-	BSSD, BEHAVE-AD (not always significant, depending on the shift)
BPC; O'Malley and Qualls (2016)	-	-	RMBPC (not always significant)
BPSD-T; Phannarus et al. (2020)	0.69	NPI	-
CDBQ; Victoroff et al. (1997)	-	-	BEHAVE-AD (0.46), CUSPAD (0.52)
CPCE; Resnick et al. (2018)	-	-	CMAI (0.44), CSDD (0.38)
DAIR; Strauss and Sperry (2002)	-		CERAD/BRSD (ns)
DBDS; Baumgarten et al. (1990)	-	-	BMDS (0.73)
DBRI; Molloy et al. (1991)	-	-	BPC (0.71)
DSSS; Loreck et al. (1994)	-	-	BEHAVE-AD (0.62-0.93), CSDD (0.84), CUSPAD (0.49-0.73)
HBS; Draper et al. (2002)	-	-	BEHAVE-AD (0.68)
IQ; Craig et al. (2008)	-	-	IAS (0.37)
NHBPS; Ray et al. (1992)	-	-	CMAI (0.91)
NPI; Cummings et al. (1994)	-	-	BEHAVE-AD (0.33-0.77)
PEAR; Jao et al. (2016)	0.71 (PEAR-apathy)	NPI-apathy	-
RAID; Shankar et al. (1999)	-	-	CSDD (0.69)
SDI; Tractenberg et al. (2003)	0.34	NPI	-

Only scales for which correlation data are available are shown.

### 3.7. New approaches: evaluate, treat, and assess

A common problem of the first pen-and-paper scales is the lack of systematic trigger assessment, which limits the ability of the clinician to tailor non-pharmacological interventions to the specific caregiver–patient dyad. Since the use of antipsychotics should be limited in light of the FDA black box warning, current guidelines recommend functional analysis-based non-pharmacological interventions as a first line of treatment for BPSD. These are largely based on modifications of the ABC algorithm (antecedent—behavior—consequence) and include a first phase when contextual data and triggering factors are gathered, a description of the target behavior is provided, and an evaluation of what happens after the behavior occurs, including, for instance, caregiver reactions (Kales et al., 2017; Dyer et al., 2018).

The first phase was generally neglected during the early stages of the BPSD scale development in the 20th century, although some examples of it exist. For instance, CABOS, an observational system to study disruptive vocalizations in NH, included data on location, activity, social environment, sound, and the use of physical restraint to orient treatment (Burgio et al., 1994). Even though the hardware and software described in the study are clearly outdated and the focus is limited to a single form of BPSD, this is perhaps the earliest example of these new approaches. The BSO-DOS system followed a few years later, and it is still quite widely used in NH in Canada, with updated manuals available. Rather than providing a score, it is a customizable and observational web-based tool that rates all the behaviors of patients, from sleeping to risk states, calculating the average episodes in terms of blocks and hours and risk. It also includes causes, planning, and interventions. All the data are analyzed and presented in the form of tables to aid in the understanding of the behavior (BSO-DOS, 2019). However, this system requires constant observation, and users require some training.

The Care Plan Checklist for Evidence of Person-Centered Approaches for BPSD (CPCE) differs in that it evaluates only those specific behaviors that are present and addressed (Resnick et al., 2018). This checklist may help explore possible neglected non-pharmacological interventions, but it is nevertheless limited to some items that might be more prevalent in the NH environment and do not take into account the severity of the behavior. The Assessment of the Environment for Person-Centered Management of BPSD and Assessment of Policies for Person-Centered Management of BPSD (AEPC), subsequently developed by the same group, completely forgoes the BPSD assessment, focusing instead on the implementation of policies and management strategies to prevent and minimize the impact of the behaviors in NH (Resnick et al., 2020). A pen-and-paper scale that combines a standard BPSD rating approach with the assessment of environmental factors is the PEAR. It has two subscales to evaluate apathy and environment, with scoring rules to guide attribution of points (Jao et al., 2016). While it is quicker than the previous instruments, it still requires some training, and its use is limited to the management of apathy in NH.

Two very similar theoretical systematizations of these new approaches have recently been proposed, which have the advantage of being implementable in a variety of settings, including home. The first is the DICE approach, which includes four different phases: Describe, Investigate, Create, and Evaluate. In the Describe phase, the caregiver is asked to “play back” the BPSD as if in a movie to let the clinicians understand and categorize the particular type of behavior rather than leaving the caregiver to define it. Recording of contextual factors in logs or diaries to be compiled at home is encouraged. The Investigate phase concerns the exclusion of potentially modifiable causes (including medical conditions), a strategy inherited from delirium management strategies, taking into account individual, caregiver, and environmental considerations. The Create step involves brainstorming with caregivers to ideate non-pharmacological treatment plans; this is followed by the Evaluate phase in which the efficacy and implementation of the interventions was evaluated (Kales et al., 2014).

Another very similar approach is the DATE algorithm, based on interprofessional Swiss recommendations. It includes the following steps, which are analogous to those of the DICE approach: Describe and measure, Analyze, Treat, and Evaluate (Tible et al., 2017). Both frameworks include the possibility of reverting to pharmacotherapy in case of potentially dangerous behaviors. The WeCareAdvisor platform is based on the DICE approach and was developed after a series of focus groups with caregivers. It is a customizable web-based tool which requires structured inputs from the users on a broad range of BPSD. It also allows for the presence of unstructured notes and feedback, includes a glossary and educational tools, and evaluates adherence to treatment. It is intended to provide caregivers with algorithm-based tailored solutions for a broad range of BPSD-related issues that they might face at home, while still warning them to seek medical advice in potentially dangerous situations (Kales et al., 2017). While it is certainly interesting, clinical trials of this system are currently ongoing, and therefore, whether it will lead to a reduction of BPSD or caregiver distress remains unknown. It also does not completely eliminate caregiver recall bias, and it still requires some training, access to a computer, and time, which might make it unsuitable for some caregivers. Moreover, the precise treatment algorithm is not published, which limits the generalizability of future results.

Although technology is at present extremely pervasive, it seems that the field of BPSD assessment has not fully profited from its potential. While it is not uncommon to observe caregivers showing video recordings of patients' behaviors, video analysis has rarely been considered as a source of information and mostly in NH. Indeed, a recent meta-analysis found that only 10 studies used video recordings to assess BPSD in NH, and the vast majority of studies relied on them to score already available scales, such as the PAS (Rosen et al., 1994) or the RTC-DAT (Mahoney et al., 1999); only in two cases did the authors develop a coding scheme for the behaviors exhibited by NH residents (Kim et al., 2019). While the use of video recordings in the home setting could have important ethical implications and could theoretically alter the behaviors of the patient and the caregiver (Moore et al., 2013), it might still be worth exploring the direct observation of BPSD with the aid of technological resources.

Finally, telemedicine approaches might be a feasible and useful method of treating BPSD, either with standard telephone-based assessment and interventions (Nkodo et al., 2022), or using artificial intelligence or dedicated mobile apps. An example of the latter is currently being studied in a registered clinical trial (Braly et al., 2021).

### 3.8. Actigraphy and biomarkers of BPSD

While it may seem surprising to talk about biomarkers of BPSD, given the importance of the setting and context, it is possible that some specific behaviors, rather than the whole spectrum of BPSD, could be captured and quantified by objective measures and surrogate markers.

Bearing in mind that caregiver report bias is a major problem, it is not surprising that research efforts have attempted to define more “objective” markers of BPSD, directly measurable on the patient. While direct clinical observation of the patient by the treating physician could be more than adequate for this purpose, it is well known that patients' behavior can change dramatically during outpatient visits, giving misleading information. Therefore, researchers have attempted to not only define surrogate markers for selected BPSD, mainly by analyzing biological fluids, such as blood or CSF (Bloniecki et al., 2014; Tumati et al., 2022), but also through different biosignals, including neurophysiological (Yang et al., 2013) or autonomic system-related ones (Suzuki et al., 2017).

Prolonged recording by wearable devices could also represent an interesting opportunity to gain insights into the real triggers of BPSD and hence to improve treatment. One of the first such attempts is actigraphy, which is used mainly, but not solely, for assessing aberrant sleep behaviors (Ancoli-Israel et al., 1997; Nagels et al., 2006; Fukuda et al., 2022).

Globally, these surrogate markers have not yet attained clinically useful status due to difficulties defining specific associations with selected behavioral phenotypes and generating meaningful cutoff values. Nevertheless, the amount of data available in the literature is quite significant and deserves a full discussion in a dedicated paper (manuscript in preparation).

### 3.9. Indications for future research

While a large number of tools are available to the clinician to evaluate BPSD, it is extremely hard to establish which instruments are the best. Each tool has advantages and disadvantages that depend on the setting, the expertise required, and the people involved. We hope to have provided clinicians with enough data to decide which instrument would be the most appropriate to address their clinical problem when managing BPSD.

New scales and tools are constantly being developed, probably because, despite more than four decades of research, results in the BPSD field remain unsatisfactory. It may therefore be worth considering which features a new instrument should have to be useful and successful. Based on the data provided here, it is clear that a new instrument should be short, easy to understand and use, require little to no training (Kales et al., 2017), and be adapted to the specific setting for which it is intended. It should also be treatment oriented, including triggers and contextual factors, evaluating both frequency and severity, and possibly providing data to clinicians. There should be a strategy to avoid caregiver recall bias, possibly through the use of technologies that allow timely online recording of BPSD. Attention should also be given to cross-cultural options and free availability of the tool to ensure its adoption in our increasingly diverse modern world. A good option would also be to make it customizable to allow for the evaluation of BPSD that need to be addressed.

Finally, for broad instruments, a global score is of limited usefulness, and we would advise against the use of statistics such as Cronbach's alpha to evaluate internal consistency. If a score is considered useful, an interpretation of it should be provided.

Our work also has implications for the concept of BPSD itself. Some models seek to explain the occurrence of BPSD within a common theoretical framework, but even these can only explain a fraction of the extreme variability in behavior (Nagata et al., 2022). As an umbrella term for a disparate and possibly ill-defined set of heterogeneous behavioral alterations, BPSD can most usefully be considered together when there is a possibility of addressing them effectively. This may account for the fact that research on BPSD is moving away from psychometric and toward tailored intervention-oriented approaches. With this practical goal in mind, we believe that drawing together different symptoms and signs under the label of BPSD still has value. However, we should consider psychometrics to be useful mainly in the context of narrow instruments, as this could still have important implications for clinical practice.

## 4. Limitations

Our study is the result of a significant effort to locate the different approaches and instruments used to assess BPSD, and as such, it has some limitations. First of all, albeit comprehensive, this is not a systematic review, and we cannot exclude the possibility that some interesting tools have been left out. Readers can ask to contribute to a Google sheet containing the data we have collected so far by following this link: https://tinyurl.com/bpsdinstruments. Ideally, this could represent a resource to inform clinicians of new approaches that will be available after the publication of our paper.

Second, estimating the real impact of the instruments is clearly difficult, and our method of counting citations and mentions in the abstract of citing papers only provides a rough estimate of the influence of the tools in research. A broader estimation of the impact on clinical practice should ideally rely on a survey or other methods of capturing how favorably clinicians regard the instrument, which could possibly include both awareness of the existence of the tools and their usage.

Finally, our approach to translations does not rule out the possibility that we have overlooked translations into some languages. We would be grateful if readers who are aware of further translations could contribute to the Google sheet previously identified.

## 5. Conclusions

In this review, we have described the characteristics of a comprehensive number of instruments to assess BPSD in terms of items, setting, target population, usage, reliability, and theoretical approach. There is a high heterogeneity among different tools, and while the NPI is probably the current gold standard, specific scales may be more suitable for particular settings. Every instrument has some limitations, and there is still a need to develop better tools for evaluating BPSD and guiding their management in clinical practice. We suggest that psychometrically sound scales should only be retained within narrow instruments to assess particular behaviors and that there should be a move toward intervention-oriented approaches when evaluating the total burden of BPSD in a patient.

## Author contributions

The process of searching, screening, and appraising the relevant studies has been performed by FP and LC. The manuscript has been drafted by FP and LT and critically revised and commented upon by all authors. All authors contributed to the article and approved the submitted version.
